# Long-Term Survival in Patients with Post-Operative Atrial Fibrillation after Cardiac Surgery: Analysis from a Prospective Cohort Study

**DOI:** 10.3390/jcdd8120169

**Published:** 2021-12-01

**Authors:** Jacopo Marazzato, Sergio Masnaghetti, Roberto De Ponti, Paolo Verdecchia, Federico Blasi, Sandro Ferrarese, Monica Trapasso, Antonio Spanevello, Fabio Angeli

**Affiliations:** 1 Department of Medicine and Surgery, University of Insubria, 21100 Varese, Italy; jacopo.marazzato@uninsubria.it (J.M.); roberto.deponti@uninsubria.it (R.D.P.); federico.blasi@uninsubria.it (F.B.); sandro.ferrarese@uninsubria.it (S.F.); antonio.spanevello@uninsubria.it (A.S.); 2 Department of Medicine and Cardiopulmonary Rehabilitation, Maugeri Care and Research Institute, IRCCS, 21049 Tradate, Italy; sergio.masnaghetti@icsmaugeri.it; 3 Fondazione Umbra Cuore e Ipertensione-ONLUS and Division of Cardiology, Hospital Santa Maria della Misericordia, 06100 Perugia, Italy; verdecchiapaolo@gmail.com; 4 Dipartimento di Igiene e Prevenzione Sanitaria, PSAL, Sede Territoriale di Varese, ATS Insubria, 21100 Varese, Italy; montrapasso@gmail.com

**Keywords:** atrial fibrillation, post-operative atrial fibrillation, cardiac surgery, oral anticoagulation, chronic disease

## Abstract

Background: Post-operative (POP) atrial fibrillation (AF) is frequent in patients who undergo cardiac surgery. However, its prognostic impact in the long term remains unclear. Methods: We followed 1386 patients who underwent cardiac surgery for an average of 10 ± 3 years. According to clinical history of AF before and after surgery, four subgroups were identified: (1) patients with no history of AF and without episodes of AF during the first 30 days after surgery (control or Group 1, n = 726), (2) patients with no history of AF before surgery in whom new-onset POP AF was detected during the first 30 days after surgery (new-onset POP AF or Group 2, n = 452), (3) patients with a history of paroxysmal/persistent AF before cardiac surgery (Group 3, n = 125, including 87 POP AF patients and 38 who did not develop POP AF), and (4) patients with permanent AF at the time of cardiac surgery (Group 4, n = 83). All-cause mortality was the primary outcome of the study. We tested the associations of potential determinants with all-cause mortality using univariable and multivariable statistical analyses. Results: Overall, 473 patients (34%) died during follow-up. After adjustment for multiple confounders, new-onset POP AF (hazard ratio (HR) = 1.31, 95% confidence interval (CI): 0.90–1.89; *p* = 0.1609), history of paroxysmal/persistent AF before cardiac surgery (HR = 1.33, 95% CI: 0.71–2.49; *p* = 0.3736), and permanent AF (Group 4) (HR = 1.55, 95% CI 0.82–2.95; *p* = 0.1803) were not associated with a significantly increased risk of mortality when compared with Group 1 (patients with no history of AF and without episodes of AF during the first 30 days after surgery). In new-onset POP AF patients, oral anticoagulation was not associated with mortality (HR = 1.13, 95% CI: 0.83–1.54; *p* = 0.4299). Conclusions: In this cohort of patients who underwent different types of heart surgery, POP AF was not associated with an increased risk of mortality. In this setting, the role of long-term anticoagulation remains unclear.

## 1. Introduction

Post-operative atrial fibrillation (POP AF) is the most common cardiac arrhythmia occurring after cardiac surgery, ranging from 15% to 36% of post-surgical cases depending on the type of surgery and the prevalence of cardiovascular and non-cardiovascular comorbidities in different patient cohorts [1,2]. However, despite being common and associated with significant intensive-care and in-hospital stay (1), the impact of POP AF on long-term survival is not clear [1,3]. Moreover, recent studies in the field have mainly investigated the long-term outcome associated with POP AF occurring in patients naïve to AF history (new-onset POP AF) and after coronary artery bypass surgery (CABG) only [1,2]. Conversely, few data are available for other different surgical settings, such as valvular heart surgery (VHS), and for POP AF episodes occurring in patients with known AF history before cardiac surgery. Even more interestingly, it seems that POP AF underpins a kaleidoscope of different pathophysiological and clinical substrates in these patients which might yield a variable long-term prognosis in specific POP AF population subsets [4].

In a prospective and wide patient cohort, the aim of this study was to investigate the long-term survival in patients presenting with POP AF after a variety of cardiac surgical procedures.

## 2. Materials and Methods

From January 2005 to May 2009, all patients undergoing a full cardiovascular rehabilitation and therapy optimization program after cardiac surgery were enrolled at our study center—Department of Medicine and Cardiopulmonary Rehabilitation, Maugeri Care and Research Institutes of Tradate, Varese, Italy. We considered a broad spectrum of surgical procedures which included coronary artery bypass (CABG), valvular heart surgery (VHS), or a combination of both, cardiac transplantation, and other minor procedures, including surgery for heart failure.

Upon admission and during the whole hospitalization, clinical and laboratory data were collected for each patient by investigators at the study site level. All clinical evaluations were performed by the attending physicians during the clinical interview and through examination of medical records. Cardiovascular and non-cardiovascular comorbidities were recorded together with the complications that eventually occurred during the in-hospital stay. Comorbidities (including type II diabetes (DM), chronic kidney disease (CKD), hypertension (HTN), coronary artery disease (CAD), and heart failure (HF)) and history of AF were defined according to current Guidelines [5,6,7,8,9,10]. Before discharge, all patients underwent a full echocardiographic assessment, and their functional capacity was tested by the six-minute walking test (6-MWT). Data related to the dispensed cardiovascular medications were also considered, including vitamin k antagonists (VKA), antihypertensive drugs (angiotensin converting enzyme inhibitors and angiotensin receptor blockers, ACE-I and ARB, respectively), beta-blockers, antiarrhythmic drugs, and digoxin. 

The primary study endpoint was death from any cause during follow-up. In the investigated population, each collected clinical variable was tested to assess its impact on outcome. In particular, to assess the long-term prognostic role of AF in the investigated patients, the original patient population was stratified into four different study subgroups according to the clinical history of this cardiac arrhythmia before and after surgery: (1) patients with no history of AF and without episodes of AF during the first 30 days after surgery (control or Group 1), (2) patients with no history of AF before surgery in whom new-onset POP AF was detected during the first 30 days after surgery (new-onset POP AF or Group 2), (3) patients with a history of paroxysmal/persistent AF before cardiac surgery (Group 3), and (4) patients with permanent AF at the time of cardiac surgery (Group 4). A sub-analysis was also performed to appraise the overall long-term survival associated with POP AF, which accounted for both post-surgical AF in patients naïve to AF history (new-onset POP AF) and those clinical cases with known AF, but in SR at the time of the surgical procedure, who developed POP AF after cardiac surgery. POP AF was defined as any AF occurring within the first 30 peri-operative days [3] and clinically detected by means of a 12-lead electrocardiogram during routine assessment. 

Finally, we investigated whether long-term anticoagulation was associated with mortality in POP AF patients. CABG patients were extracted from the general patient population and then sub-analyzed to account for the potential confounding role of VHS on the long-term outcome, thus avoiding any potential bias connected with the use of anticoagulation in patients with mechanical prostheses in addition to POP AF. Oral anticoagulants were administered in this patient group according to clinical judgment.

This study conforms to the Declaration of Helsinki on human research and was approved by the Ethical Committee of ICS Maugeri (approval No. 2483CE). Patients or their legal guardian signed informed consent.

Statistical analyses were performed using Medcalc^®^, version 20.009 (MedCalc Software Ltd., Ostend, Belgium), Stata, version 16 (StataCorp LP, College Station, TX, USA), and R, version 4.1.2 (R Foundation for Statistical Computing, Vienna, Austria). We expressed continuous variables as mean ± standard deviation (SD) and the categorical variables as proportions. We analyzed differences in proportions between groups using the χ^2^ test. Mean values of variables were compared by the independent sample *t*-test or analysis of variance, when appropriate.

For survival analyses, event-free curves were estimated using the Kaplan–Meier product-limit method and compared by the Mantel (log-rank) test. We evaluated the effect of prognostic factors on mortality by the Cox proportional-hazards model, and the derived hazard ratios (HRs) were expressed with their corresponding two-sided 95% confidence intervals (CI). To evaluate the independent prognostic impact of AF, we added the four different study subgroups (as defined by the clinical history of AF before and after surgery) to a multivariable model including covariates which yielded independent statistical significance. To include predictors in the multivariable model, we used Akaike’s information criterion (AIC) and the Bayesian information criterion (BIC) to compare different multivariable models based on their fit to the data. 

Analyses were performed using a significance level of α = 0.05 (2-sided).

## 3. Results

### 3.1. Overall Patient Population

One thousand three hundred and eighty-six (1386) patients were prospectively enrolled at our study center. The clinical features of the overall patient population are reported in Table 1. The prevalence of comorbidities was remarkable in this generally elderly population (65 ± 12 years). Prevalence of HTN, type II DM, and CAD was 65%, 21%, and 64%, respectively. History of paroxysmal/persistent or permanent AF before surgery was known in 125 (9%) and 83 (6%) of the patients, respectively. Moreover, a non-negligible proportion of patients reported prior history of HF (8%) and chronic obstructive pulmonary disease (COPD) (7%). As for the type of heart surgery, most of the patients underwent CABG (47%), VHS (36%), or a combination of both (12%). Among post-surgical complications, infections (34%), acute renal failure (11%), and deep vein thrombosis (11%) were most frequently observed. At discharge, a large proportion of patients were treated with VKA oral anticoagulants (55%), renin-angiotensin system blockers (63%), and beta-blockers (73%). Of note, despite a generally good left ventricular ejection fraction documented on the echocardiogram (54% ± 10%), in most cases, the functional capacity tested by the 6-MWT was suboptimal on average (426 ± 113 m). The mean in-hospital stay was 21 ± 7 days.

### 3.2. POP AF Population

POP AF was the most common post-surgical complication in the investigated population (n = 539, 39%), accounting for both new-onset episodes (n = 452, 84%) and POP AF cases occurring in patients with known AF before cardiac surgery (n = 87, 16%).

Demographic, clinical, and periprocedural features of POP AF cases compared with patients without history of AF and who never developed AF after surgery (non-POP AF or controls) are displayed in Table 2. Compared with controls, POP AF patients were generally older, suffered from more cardiovascular and non-cardiovascular comorbidities, had more cases of post-surgical complications, and generally showed longer in-hospital stay and a lower functional capacity at discharge (Table 2).

According to the clinical history of AF before and after surgery, the following four subgroups were identified (see Methods Section): (1) patients with no history of AF and without episodes of AF during the first 30 days after surgery (control or Group 1, n = 726), (2) patients with no history of AF before surgery in whom new-onset POP AF was detected during the first 30 days after surgery (new-onset POP AF or Group 2, n = 452), (3) patients with a history of paroxysmal/persistent AF before cardiac surgery (Group 3, n = 125, including 87 POP AF patients and 38 who did not develop POPAF), and (4) patients with permanent AF at the time of cardiac surgery (Group 4, n = 83).

### 3.3. Univariable Analysis: The Long-Term Prognostic Impact of Atrial Fibrillation

In the study population, the overall mortality rate was 34% over a long-term follow-up (10 ± 3 years, short-term follow-up less than 1 year in roughly 2% of patients only). Table 1 displays all the clinical and laboratory variables associated with a poor long-term prognosis in this post-surgical population.

As reported in Table 1, in addition to age (HR: 1.09, 95% CI: 1.08–1.10, *p* < 0.0001) and the type of surgery (VHS when combined with CABG: HR: 2.06, 95% CI: 1.61–2.64, *p* < 0.0001), a variety of post-surgical complications and cardiovascular and non-cardiovascular comorbidities were associated with a worse clinical outcome (Table 1). In particular, the long-term risk of all-cause death was more than doubled in patients with a prior history of hospitalizations for HF (HR: 2.21, 95% CI: 1.70–2.87, *p* < 0.0001) and roughly tripled in those who developed post-operative acute renal failure during the in-hospital stay (HR: 2.97, 95% CI: 2.38–3.71, *p* < 0.0001).

**Figure 1:**
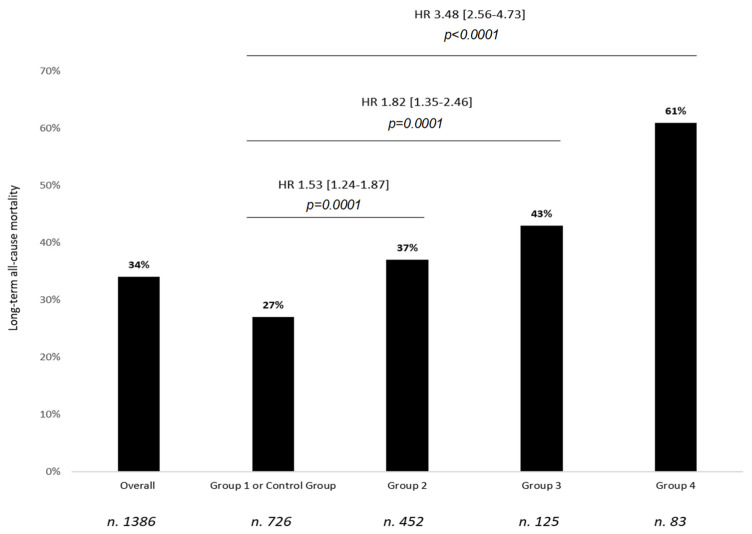
Overall long-term survival and prognostic differences between the investigated study subgroups. Group 1: patients who never developed AF (control), Group 2: patients with no history of AF before surgery in whom POP AF was clinically detected (new-onset POP AF), Group 3: patients with pre-surgical history of paroxysmal/persistent AF, and Group 4: permanent AF patients. A raw estimate of long-term all-cause mortality is reported for each study subgroup. Comparisons between different study subgroups are reported as hazard ratios (HR) and related 95% confidence intervals [squared brackets], and *p*-values as assessed in the univariable analysis. Corrections for multiplicity did not significantly change confidence intervals for comparisons. Number of patients for each study group is reported below each bar. POP AF: post-operative atrial fibrillation.

**Figure 2:**
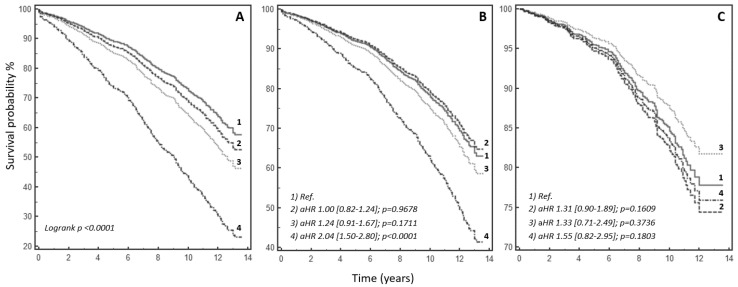
Survival analysis. (**A**) Kaplan–Meier survival curves for study subgroups. (**B**) Age-adjusted survival curves (Cox proportional-hazards model). (**C**) Fully adjusted (for all the clinical variables which proved statistically significant in the multivariable model) survival curves (Cox proportional-hazards model). Legend: (1) patients who never developed AF (control group), (2) patients with no history of AF before surgery in whom POP AF was clinically detected (new-onset POP AF), (3) patients with pre-surgical history of paroxysmal/persistent AF, and (4) permanent AF patients. Comparisons between different study subgroups are reported as hazard ratios (HR) and related 95% confidence intervals [squared brackets], and *p*-values as assessed in both univariable and multivariable analyses. POP AF: post-operative atrial fibrillation.

As displayed in Figure 1, when the history of AF was considered, compared with controls (patients with no history of AF and without episodes of AF during the first 30 days after surgery, Group 1, n = 726), patients in Group 2 (HR: 1.53, 95% CI: 1.24–1.87, *p* = 0.0001), Group 3 (HR: 1.82, 95% CI: 1.35–2.46, *p* = 0.0001), and Group 4 (HR: 3.48, 95% CI: 2.56–4.73, *p* = 0.0001) were all significantly associated with a worse long-term prognosis (Figure 2A). Rates of all-cause mortality were 27%, 37%, 43%, and 61% for Group 1, Group 2, Group 3, and Group 4, respectively. When compared with controls (Group 1), the unadjusted increased risks of long-term all-cause mortality were 37%, 45%, and 61% for Group 2, Group 3, and Group 4 respectively (*p* < 0.0001) (Figure 1), with consistent results at survival analysis (log-rank *p* < 0.0001) (Figure 2A). Similar results were also observed for the entire cohort of POP AF patients (both post-surgical AF in patients naïve to AF history and cases with known AF, but in SR at the time of the surgical procedure developing POP AF after cardiac surgery; n = 539): POP AF displayed an increased unadjusted risk of long-term all-cause mortality when compared with cases who never developed AF before and/or after heart surgery (controls or Group 1) (HR: 1.59, 95% CI: 1.30–1.93, *p* < 0.0001). These results were consistent for patients with (HR: 1.53, 95% CI: 1.25–1.89, *p* < 0.0001) and without a history of AF before cardiac surgery (Supplementary Figure S1A).

### 3.4. Multivariable Analysis: Relationship between Atrial Fibrillation and Other Prognostic Variables over a Long-Term Follow-Up

To investigate the independent prognostic impact of AF in the appraised post-surgical population, we developed a multivariable survival model (Table 3) adding all covariates with independent prognostic value (selected among those with documented statistical significance at univariable analyses, Table 1).

Although permanent AF was still associated with a worse clinical outcome after adjustment for age (HR: 2.04, 95% CI: 1.50–2.80, *p* < 0.0001; Figure 2B), when fully adjusted for all the covariates included in the multivariable model (Table 3), neither new-onset POP AF (Group 2) (HR: 1.31, 95% CI: 0.90–1.89, *p* = 0.1609), nor history of paroxysmal/persistent AF before cardiac surgery (Group 3) (HR: 1.33, 95% CI: 0.71–2.49, *p* = 0.3736), nor permanent AF (Group 4) (HR: 1.55, 95% CI: 0.82–2.95, *p* = 0.1803) were significantly associated with the risk of long-term mortality (Figure 2C). No significant interactions between covariates were documented (all *p* > 0.05).

Similarly, POP AF was no longer associated with a dismal long-term survival after adjustment for age (HR: 1.01, 95% CI: 0.83–1.24, *p* = 0.9054) or for the covariates included in the final multivariable model (HR: 1.18, 95% CI: 0.81–1.72, *p* = 0.3817) when compared to patients who never developed this post-surgical arrhythmia (controls or Group 1). Consistent results were observed for the investigated POP AF subgroups (Supplementary Figure S1B,C)

Most of the covariates which displayed an independent risk of long-term all-cause mortality in the multivariable model were fairly common in patients with AF history. As compared with controls (Group 1), patients in Groups 2, 3, and 4 were significantly older (*p* < 0.001), reported more hospitalizations for HF (*p* < 0.001), exacerbations for COPD (*p* = 0.0001), higher values of serum creatinine documented during the cardiac rehabilitation program (*p* < 0.001), and finally, a generally suboptimal 6-MWT at discharge (*p* < 0.001) (Supplementary Table S1). Similar results were observed for POP AF patients (Table 2).

### 3.5. Prognostic Impact of Oral Anticoagulation Administered at Discharge in Patients with New-Onset Post-Surgical Atrial Fibrillation

**Figure 3:**
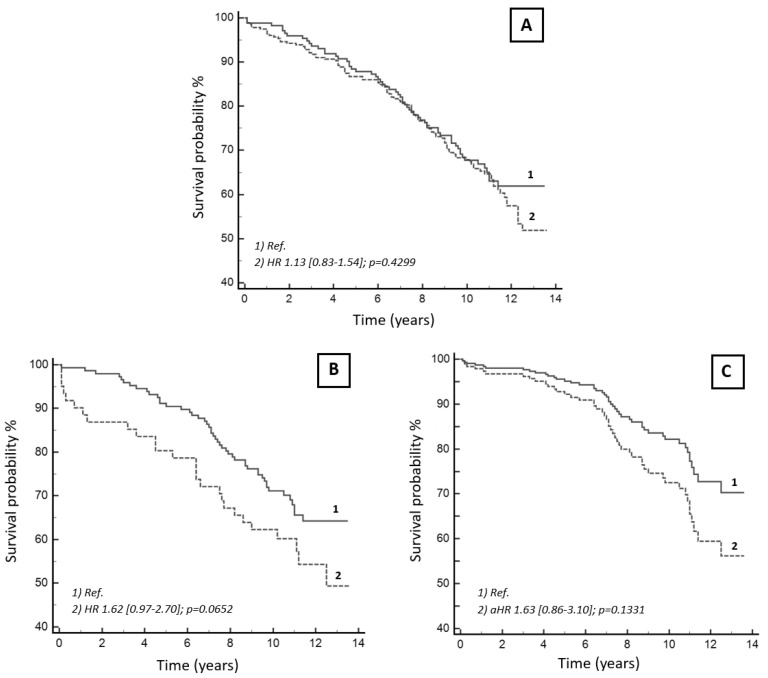
Long-term survival in patients with new-onset post-surgical atrial fibrillation on/off oral anticoagulation after hospital discharge. (**A**) Unadjusted survival curves in patients with new-onset post-operative atrial fibrillation occurring after all types of cardiac surgery. (**B**) Unadjusted survival Kaplan–Meier curves in patients who underwent coronary artery bypass surgery only. (**C**) Fully adjusted curves in this latter population. Legend: Not anticoagulated patients with new-onset post-operative atrial fibrillation (1) and anticoagulated patients (2). Comparisons between the two subgroups are reported as hazard ratios (HR) and related 95% confidence intervals [squared brackets], and *p*-values as assessed in both univariable and multivariable analyses. See text for details.

As observed in the Kaplan–Meier curves reported in Figure 3A, compared with patients with new-onset POP AF (Group 2) without oral anticoagulants at hospital discharge (n = 173/452, 38%), VKA oral anticoagulation (n = 279, 62%) did not show any significant association with mortality during follow-up (HR = 1.13 (95% CI 0.83–1.54), *p* = 0.4299). However, when the post-CABG subgroup was uniquely considered (n = 208, 46%), differently from patients off oral anticoagulants, a non-significant trend towards a worse clinical outcome was observed in Group 2 patients dispensed early with oral anticoagulants (HR: 1.62, 95% CI: 0.97–2.70, *p* = 0.0652) (Figure 3B), even after adjustment for all demographic and clinical variables included in the investigated multivariable model (HR: 1.63, 95% CI: 0.86–3.10, *p* = 0.1331) (Figure 3C).

## 4. Discussion

In this large prospective patient cohort undergoing different cardiac surgical procedures, we showed that POP AF is a frequent post-surgical complication connected with an unadjusted higher risk of long-term mortality. However, when this hazard was adjusted for different demographic, clinical, and surgical covariates, both new-onset POP AF and POP AF in patients with a pre-surgical history of AF were no longer associated with a worse survival over a long-term follow-up. Moreover, the role of long-term anticoagulation in this setting remains unclear.

Over the last decades, the long-term prognostic impact of POP AF has been debated since similarly designed studies have reported different long-term survival data in patients undergoing cardiac surgery [11,12]. Three systematic reviews and meta-analyses [1,2,13] including up to 239,018 surgical patients [1] have recently endeavored to summarize the available clinical evidence in the field. Compared with patients without POP AF, a significantly higher risk of long-term all-cause mortality (19–29% on average) [1,13] and stroke (4%) [1,2,13] has been reported for those developing POP AF after CABG [2] or VHS [1,13], even after adjustment for age, sex, body mass index, and major comorbidities [2,13]. However, these pooled survival estimates were affected by a significant inter-study heterogeneity, partly explained by variable arrhythmia and outcome definitions [2,13]. Indeed, POP AF was considered for either 30 min or longer episodes [1], requiring [14] or not requiring [15] any medical intervention and occurring within 10 days [15] or 3 months [16] after surgery. On the other hand, exclusion of deaths occurring within 1–3 months after surgery may have resulted in a biased estimate of the long-term survival reported by some evidence [12,16]. Not least, some studies would suggest that different clinical forms of POP AF (i.e., new-onset POP AF vs. POP AF in patients with known AF before surgery) may portend a different long-term outcome (4), hinting at the existence of different prognostic families of POP AF in this complex scenario. To further complicate matters, two Swedish registries [3,17] not included in these meta-analyses [1,2,13] have recently reported different results in this post-surgical population.

In a vast observational cohort study including 24,523 patients, Taha et al. showed how new-onset POP AF occurring after isolated CABG was associated with an adjusted increased risk of ischemic stroke (HR: 1.18, 95% CI: 1.05–1.32), HF hospitalizations (HR: 1.35, 95% CI: 1.21–1.51), and recurrent AF (HR: 4.16, 95% CI: 3.76–4.60), but not with all-cause mortality (HR: 1.08, 95% CI: 0.98–1.18) over a long-term follow-up (median 4.5 years, range 0–9 years) [3]. Similar results were reported by Thoren et al. in a comparable patient population [17]. Despite the huge number of included patients and the prospective nature of these registries, no data are available for patients undergoing VHS, cardiac transplantation, or cardiac surgery for the correction of heart failure. Furthermore, by analyzing patients with new-onset POP AF only, the prognostic influence of a history of AF before cardiac surgery has not been explored by these studies.

To the best of our knowledge, this is the first long-term prospective study to have clearly shown the absence of any long-term impact on the risk of all-cause mortality of POP AF occurring after any type of cardiac surgery. Older age, history of HF and COPD before surgery, and higher post-operative levels of serum creatinine were the only demographic and clinical variables which proved to exert a long-term prognostic impact after heart surgery, with no significant impact regarding any type of AF history before and/or after surgery. This suggests that POP AF—and more generally AF as a nosological entity—may act as a prognostic confounder in a surgical population made up of generally old patients suffering from several concomitant cardiovascular and non-cardiovascular morbidities. On the other hand, absence of structural heart disease and good functional capacity at discharge independently correlated with a better survival in our analysis.

In light of these findings, it is legitimate to wonder whether administration of oral anticoagulants in patients with POP AF may improve their long-term survival, especially when no history of this arrhythmia is known before cardiac surgery (new-onset POP AF).

Although some studies suggest a non-negligible thromboembolic profile in this population [18,19], Taha et al. [3] showed neither a reduced risk of all-cause death nor of ischemic stroke in patients on oral anticoagulants for new-onset POP AF after isolated CABG. Interestingly, in a similar patient cohort, we even observed a trend towards a worse long-term survival for anticoagulated patients.

Even though a significant number of life-threatening major bleedings might explain these findings [3], in consideration of the relatively small sample size of patients with isolated CABG and the unique use of VKA oral anticoagulants in the present study, no definitive conclusions can be drawn from these results. Indeed, the implementation of other different oral anticoagulants was not carried out in this study since non-VKA oral anticoagulants were not marketed at the time of patients’ enrollment in Italy nor were data regarding potential shifts from one anticoagulant to another collected during the follow-up.

Not least, all-cause death was the only outcome to be addressed in our analysis and no data regarding other prognostic events are available, such as ischemic stroke, recurrence of AF, and hospitalizations for HF. This should be regarded as the major limitation of the present study. Moreover, the effect of patients’ lifestyle modifications or control of chronic medical history after surgery on the long-term prognosis was not assessed in this study. Therefore, further prospective, and adequately designed and powered studies, are mandatory to provide us with more clear answers in this hazy clinical scenario.

## 5. Conclusions

In a large prospective patient cohort undergoing different types of heart surgery, the risk of POP AF is not negligible. However, POP AF and AF history were not independently associated with an increased risk of long-term mortality, differently from age and other known comorbidities. The usefulness of long-term anticoagulation in these patients remains unproven. These data suggest that, in this surgical population, the focus should be shifted from POP AF treatment to the accurate assessment of underlying patient comorbidities.

## Figures and Tables

**Table 1:** Demographic and clinical features of the investigated surgical patient population.

	Overall	Survived	Deceased	*p*	Univariable Analysis HR [95% CI]	*p*
**No. of patients (n, %)**	1386	913 (66)	473 (34)			
Age (years)	65 ± 12	62 ± 12	72 ± 9	<0.0001	1.09 [1.08–1.10]	<0.0001
Sex F (n, %)	419 (30)	257 (28)	162 (34)	0.0224	1.30 [1.07–1.57]	0.0072
BMI (kg/m^2^)	25 ± 5	25 ± 4	25 ± 5	0.6044	0.99 [0.97–1.01]	0.3792
HTN (n, %)	869 (65)	533 (58)	336 (71)	<0.0001	1.56 [1.28–1.90]	<0.0001
Type II DM (n, %)	290 (21)	159 (17)	131 (28)	<0.0001	1.66 [1.35–2.02]	<0.0001
Prior history of CAD (n, %)	894 (64)	576 (63)	318 (67)	0.039	1.09 [0.90–1.32]	0.3670
Prior history of HF (n, %)	116 (8)	52 (6)	64 (13)	<0.0001	2.21 [1.70–2.87]	<0.0001
LVEF (%)	54 ± 10	55 ± 10	51 ± 12	<0.0001	0.97 [0.96–0.98]	<0.0001
COPD	91 (7)	41 (4)	50 (11)	<0.0001	2.11 [1.58–2.82]	<0.0001
**AF type before Cardiac Surgery:**						
Paroxysmal/Persistent AF	125 (9)	71 (8)	54 (11)	<0.0001	1.82 [1.35–2.46]	0.0001
Permanent AF	83 (6)	32 (4)	51 (11)	<0.0001	3.48 [2.56–4.73]	<0.0001
**Type of cardiac surgery (n, %):**				<0.0001		
CABG	655 (47)	457 (50)	198 (42)		Ref	
VHS	495 (36)	326 (36)	169 (36)		1.24 [1.0–1.52]	0.0413
Cardiac Transplantation	31 (2)	27 (3)	4 (1)		0.45 [0.17–1.19]	0.1097
PCI plus CABG	12 (1)	10 (1)	2 (0)		0.51 [0.13–2.05]	0.3466
CABG plus VHS	173 (12)	82 (9)	91 (19)		2.06 [1.61–2.64]	<0.0001
Others	20 (2)	11 (1)	9 (2)		1.80 [0.92–3.50]	0.0852
**POP AF**	539 (39)	331 (36)	208 (44)	0.0001	1.53 [1.26–1.85]	<0.0001
**New-onset POP AF**	452 (33)	283 (31)	169 (36)	<0.0001	1.53 [1.24–1.87]	0.0001
**POP complications:**						
Stroke	35 (3)	18 (2)	17 (4)	0.1000	1.64 [1.01–2.66]	0.0447
Myocardial Infarction	31 (2)	22 (2)	9 (2)	0.6792	0.87 [0.45–1.68]	0.6849
Acute HF	50 (4)	28 (3)	22 (5)	0.1777	1.65 [1.07-2.52]	0.0223
Acute Respiratory Failure	131 (9)	85 (9)	46 (10)	0.8779	1.10 [0.81–1.49]	0.5367
Acute renal failure	158 (11)	101 (11)	57 (12)	<0.0001	2.97 [2.38–3.71]	<0.0001
Infections	472 (34)	287 (31)	185 (39)	0.0051	1.35 [1.12–1.62]	0.0015
DVT	158 (11)	88 (10)	70 (15)	0.0055	1.43 [1.11–1.84]	0.0056
**Blood tests at admission (rehabilitation program):**						
POP Creatinine (mg/dL)	1.26 ± 0.61	1.14 ± 0.44	1.49 ± 0.81	<0.0001	1.56 [1.45–1.68]	<0.0001
POP Hemoglobin (g/dL)	11.8 ± 1.19	11.9 ± 1.19	11.7 ± 1.19	0.0417	0.91 [0.84–0.98]	0.0131
POP C-reactive protein (mg/L)	5.2 ± 4.2	4.9 ± 3.9	5.7 ± 4.7	0.0292	1.04 [1.02–1.07]	0.0014
**In-hospital stay (days)**	21 ± 7	20 ± 7	22 ± 8	<0.0001	1.03 [1.02–1.04]	<0.0001
**6-MWT at discharge (meters)**	426 ± 113	727 ± 102	340 ± 110	<0.0001	0.99 [0.98–0.99]	<0.0001
**Medications after discharge:**						
VKA oral anticoagulants	757 (55)	459 (50)	298 (63)	<0.0001	1.59 [1.32–1.91]	<0.0001
ACE-I	739 (53)	458	281	0.0013	1.36 [1.13–1.63]	0.0010
ARB	136 (10)	80	56	0.0847	1.28 [0.97–1.69]	0.0849
Beta-blockers	1010 (73)	685	325	0.0145	0.76 [0.63–0.92]	0.0055
Antiarrhythmic medications	353 (25)	220	133	0.1177	1.21 [0.99–1.48]	0.0609
Digoxin	26 (2)	17	9	0.8762	1.05 [0.55–2.03]	0.8814
**Follow-up:**						
Average follow-up (years)	10 ± 3	11 ± 1	7 ± 3	<0.0001		
Follow-up < 1 year (n, %)	29 (2)	0 (0)	29 (6)	<0.0001		

Legend. 6-MWT = 6-min walking test; ACE-I = angiotensin converting enzyme inhibitor; AF = atrial fibrillation; ARB = angiotensin receptor blockers; BMI = body mass index; CABG = coronary artery bypass surgery; CAD = coronary artery disease; COPD = chronic obstructive pulmonary disease; DVT = deep vein thrombosis; DM = diabetes mellitus; F = female; HF = heart failure; HTN = hypertension; LVEF = left ventricular ejection fraction; POP = post-operative; VHS = valvular heart surgery; VKA = vitamin K antagonists.

**Table 2:** Demographic and clinical features of POP AF patients compared with patients without AF before and after surgery.

	POP AF		*p*
	No (N = 726)	Yes (N = 539)	
Age (years)	62 ± 12	69 ± 9	<0.0001
Sex F (n, %)	172 (23)	193 (36)	<0.0001
BMI (kg/m^2^)	25 ± 5	25 ± 5	0.3693
HTN (n, %)	428 (59)	367 (68)	0.0009
Type II DM (n, %)	157 (22)	113 (21)	0.7768
Prior history of CAD (n, %)	515 (71)	333 (62)	0.0006
Prior history of HF (n, %)	45 (6)	51 (9)	0.0303
LVEF (%)	55 ± 10	53 ± 11	0.0035
COPD	27 (4)	52 (10)	<0.0001
**Type of cardiac surgery (n, %):**			<0.0001
CABG	421 (56)	217 (40)	
VHS	203 (28)	209 (39)	
Cardiac Transplantation	27 (4)	0 (0)	
PCI plus CABG	6 (1)	4 (1)	
CABG plus VHS	61 (8)	97 (20)	
Others	8 (1)	12 (2)	
**POP complications:**			
Stroke	12 (2)	20 (4)	0.0212
Myocardial Infarction	23 (3)	7 (1)	0.0308
Acute HF	24 (3)	15 (3)	0.1777
Acute Respiratory Failure	66 (9)	52 (10)	0.7365
Acute renal failure	59 (8)	79 (15)	0.0002
Infections	235 (32)	197 (36)	0.1212
DVT	71 (10)	78 (15)	0.0105
**Blood tests at admission**			
POP Creatinine (mg/dL)	1.20 ± 0.59	1.34 ± 0.62	<0.0001
POP Hemoglobin (g/dL)	12.0 ± 1.20	11.8 ± 1.16	0.0313
POP C-reactive protein (mg/L)	4.9 ± 4.1	5.7 ± 4.5	0.0043
**In-hospital stay (days)**	20 ± 7	22 ± 7	<0.0001
**6-MWT at discharge (meters)**	451 ± 107	403 ± 109	<0.0001
**Medications after discharge:**			
VKA oral anticoagulants	285 (39)	360 (67)	<0.0001
ACE-I	374 (51)	297 (55)	0.2064
ARB	62 (9)	61 (11)	0.1010
Beta-blockers	563 (78)	382 (71)	0.0069
Antiarrhythmic medications	37 (5)	294 (54)	<0.0001
Digoxin	1 (0)	11 (2)	0.0006
**Follow-up:**			
Average follow-up (years)	10 ± 3	9 ± 3	<0.0001
Follow-up <1 year (n, %)	15 (2)	9 (2)	0.6095

Legend. 6-MWT = 6-min walking test; ACE-I = angiotensin converting enzyme inhibitor; AF = atrial fibrillation; ARB = angiotensin receptor blockers; BMI = body mass index; CABG = coronary artery bypass surgery; CAD = coronary artery disease; COPD = chronic obstructive pulmonary disease; DVT = deep vein thrombosis; DM = diabetes mellitus; F = female; HF = heart failure; HTN = hypertension; LVEF = left ventricular ejection fraction; POP = post-operative; VHS = valvular heart surgery; VKA = vitamin K antagonists.

**Table 3:** Multivariable model (see text for details) investigating the independent prognostic value of atrial fibrillation.

Covariates	Comparison	HR [95% CI]	*p*
Age	1-year increase	1.08 [1.05–1.10]	<0.0001
COPD	Yes vs. No	1.72 [1.05–2.82]	0.0309
History of HF	Yes vs. No	1.79 [1.10–2.93]	0.0194
LVEF	1% increase	0.98 [0.97–0.99]	0.0448
Serum creatinine at admission	1 mg/dL increase	1.43 [1.13–1.80]	0.0026
6-MWT at discharge	1 m increase	0.98 [0.97–0.99]	0.0031
Atrial fibrillation groups (Group 1 as reference)			
Group 2	Group 1	1.31 [0.90–1.89]	0.1609
Group 3	Group 1	1.33 [0.71–2.49]	0.3736
Group 4	Group 1	1.55 [0.82–2.95]	0.1803

Legend. Group 1: patients with no history of AF and without episodes of AF during the first 30 days after surgery, Group 2: patients with no history of AF before surgery in whom new-onset POP AF was detected during the first 30 days after surgery (new-onset POP AF), Group 3: patients with a history of paroxysmal/persistent AF before cardiac surgery, and Group 4: patients with permanent AF at the time of cardiac surgery. 6-MWT = 6-min walking test; COPD = chronic obstructive pulmonary disease; HF = heart failure; LVEF = left ventricular ejection fraction.

## Data Availability

The data underlying this article cannot be shared publicly due to privacy of individuals that participated in the study. The data will be shared on reasonable request to the corresponding author.

## Supplementary Materials

The following are available online at https://www.mdpi.com/article/10.3390/jcdd8120169/s1, Figure S1: Survival analysis in POP AF patients. Table S1: Features of the investigated surgical population according to history of atrial fibrillation
