# Supplementary material for: Long-Term Survival in Patients with Post-Operative Atrial Fibrillation after Cardiac Surgery: Analysis from a Prospective Cohort Study
Source: J Cardiovasc Dev Dis. 2021 Dec 1;8(12):169. doi: 10.3390/jcdd8120169 (PMC8707624; doi:10.3390/jcdd8120169)
Supplement: Supplementary file 1 [file jcdd-08-00169-s001.zip › jcdd-1442739-SI.pdf]

Figure S1. Survival analysis in POP AF patients. A) Unadjusted Kaplan-Meier curves in the different study subgroups; B) survival curves adjusted for age; C) survival probability fully adjusted for all the clinical variables which proved statistically significant in the multivariable model. *Legend*. 1) patients who never developed AF (control group); 2) patients with no history of AF before surgery in whom POP AF was clinically detected (new-onset POP AF); 3) patients with pre-surgical history of paroxysmal/persistent AF who developed POP AF. Comparisons between different study subgroups are reported as Hazard Ratios (HR) and related 95% confidence intervals [squared brackets] and *p* values as assessed in both univariable and multivariable analyses. POP AF: post operative atrial fibrillation.

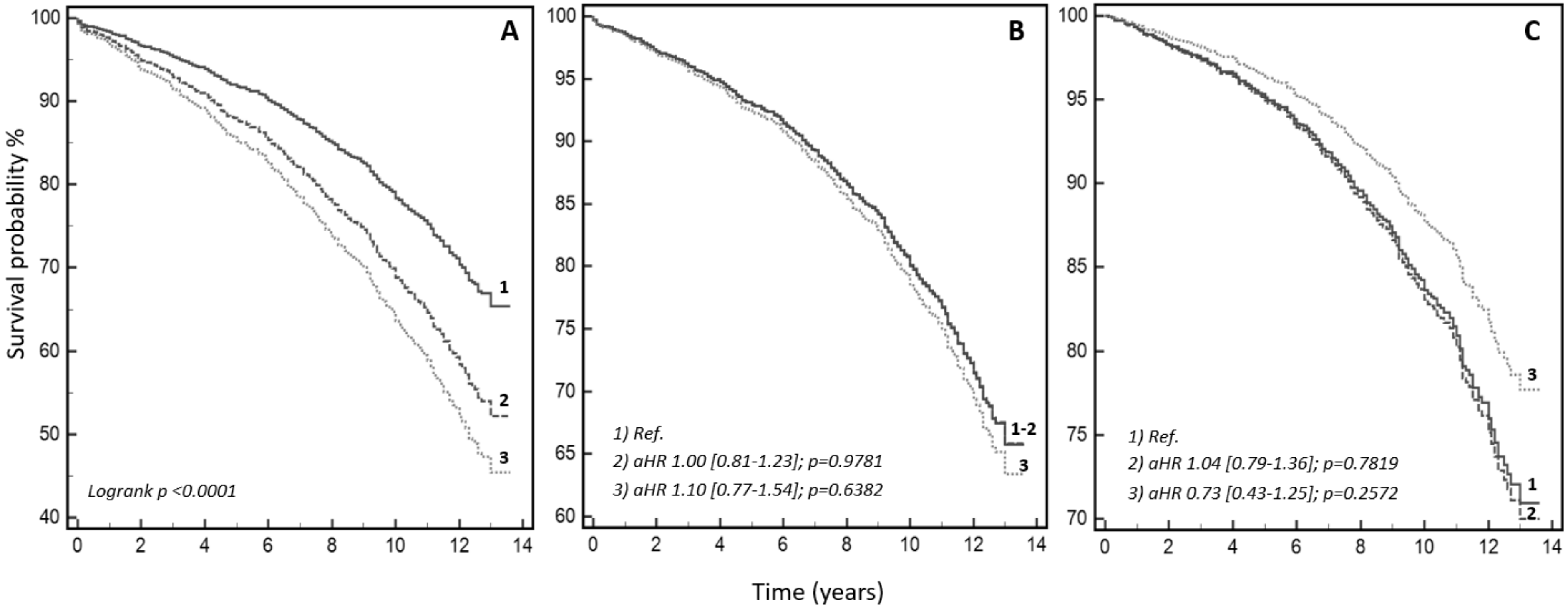

Table S1. Features of the investigated surgical population according to history of atrial fibrillation

	Overall	Control group	New-onset POP AF	Paroxysmal/persistent AF before surgery	Permanent AF	*p*
**N. patients (n, %)**	1386	726 (52)	452 (33)	125 (9)	83 (6)	
**Age (years)**	65 ± 12	62 ± 12	69 ± 9	68 ± 10	71 ± 9	<0.001
**Sex F (n, %)**	419 (30)	172 (24)	147 (32)	64 (51)	36 (43)	<0.0001
**BMI (kg/m2)**	25 ± 5	25 ± 5	25 ± 5	26 ± 5	26 ± 6	0.6530
**HTN (n, %)**	869 (65)	428 (59)	312 (69)	76 (61)	53 (64)	0.0063
**Type II DM (n, %)**	290 (21)	157 (22)	103 (23)	13 (10)	17 (20)	0.0229
**Prior history of CAD (n, %)**	894 (64)	515 (71)	301 (67)	48 (38)	30 (36)	<0.0001
**Prior history of HF (n, %)**	116 (8)	45 (6)	32 (7)	24 (19)	15 (18)	<0.0001
**LVEF (%)**	54 ± 10	55 ± 10	53 ± 10	53 ± 11	53 ± 10	0.0570
**COPD**	91 (7)	27 (4)	45 (10)	10 (8)	9 (11)	0.0001
**Type of cardiac surgery (n, %):**						
CABG	655 (47)	421 (58)	204 (45)	20 (16)	10 (12)	<0.0001
VHS	495 (36)	203 (28)	148 (33)	84 (67)	60 (72)	<0.0001
Cardiac Transplantation	31 (2)	27 (4)	0 (0)	4 (3)	0 (0)	<0.0001
PCI plus CABG	12 (1)	6 (1)	4 (1)	1 (1)	1 (1)	<0.0001
CABG plus VHS	173 (12)	61 (8)	85 (19)	15 (12)	12 (14)	<0.0001
Others	20 (2)	8 (1)	11 (2)	1 (1)	0 (0)	<0.0001
**POP complications:**						
Stroke	35 (3)	12 (2)	18 (4)	3 (2)	2 (2)	0.1043
Myocardial Infarction	31 (2)	23 (3)	7 (2)	0 (0)	1 (0)	0.0681
Acute HF	50 (4)	24 (3)	13 (3)	8 (6)	5 (6)	0.1659
Acute Respiratory Failure	131 (9)	66 (9)	49 (11)	7 (6)	9 (11)	0.3228
Acute renal failure	158 (11)	59 (8)	73 (16)	10 (8)	16 (19)	<0.0001
Infections	472 (34)	235 (32)	161 (36)	49 (39)	27 (32)	0.3962
DVT	158 (11)	71 (10)	66 (15)	16 (13)	5 (6)	0.0281
**Blood tests at admission:**						
POP Creatinine (mg/dL)	1.26 ± 0.61	1.19 ± 0.59	1.35 ± 0.63	1.29 ± 0.58	1.37 ± 0.70	<0.001
POP Hb (g/dL)	11.8 ± 1.19	11.9 ± 1.2	11.8 ± 1.1	11.7 ± 1.2	11.4 ± 1.2	0.001
POP C-reactive protein (mg/L)	5.2 ± 4.2	5 ± 4	6 ± 5	5 ± 4	4 ± 3	0.014

**In-hospital stay (days)**	21 ± 7	20 ± 7	22 ± 7	22 ± 7	24 ± 9	<0.001
**6-MWT at discharge (metres)**	426 ± 113	451 ± 107	405 ± 111	402 ± 106	367 ± 125	<0.001
**Medications after discharge:**						
VKA	757 (55)	285 (39)	279 (62)	112 (90)	81 (98)	<0.0001
ACE-I	739 (53)	374 (51)	254 (56)	63 (50)	48 (58)	0.3133
ARB	136 (10)	62 (8)	48 (11)	17 (14)	9 (11)	0.2888
Beta-blockers	1010 (73)	563 (77)	330 (73)	67 (54)	50 (96)	<0.0001
Antiarrhythmic medications	353 (25)	37 (5)	237 (52)	73 (58)	6 (7)	<0.0001
Digoxin	26 (2)	1 (0)	3 (1)	9 (7)	13 (16)	<0.0001
**Follow-up:**						
Average follow-up (years)	10 ± 3	10 ± 3	9 ± 3	9 ± 3	7 ± 4	<0.001

Abbreviations. 6-MWR= 6-minute walking test; ACE-I= angiotensin converting enzyme-inhibitor; AF= atrial fibrillation; ARB= Angiotensin Receptor Blockers; CABG= coronary artery bypass surgery; COPD= chronic obstructive pulmonary disease; DVT=deep vein thrombosis; BMI= body mass index; CAD= coronary artery disease; DM= diabetes mellitus; LVEF= left ventricular ejection fraction; HF= Heart Failure; HTN=hypertension; F=female; VHS=valvular heart surgery; VKA= vitamin-K antagonists.
